# Exact Decomposition of Optimal Control Problems via Simultaneous Block Diagonalization of Matrices

**DOI:** 10.1109/ojcsys.2022.3231553

**Published:** 2022-12-22

**Authors:** AMIRHOSSEIN NAZERIAN, KSHITIJ BHATTA, FRANCESCO SORRENTINO

**Affiliations:** 1 Mechanical Engineering Department, University of New Mexico, Albuquerque, NM 87131 USA; 2 Mechanical and Aerospace Engineering, University of Virginia, Charlottesville, VA 22903 USA

**Keywords:** Decoupling, optimal control, simultaneous block diagonalization

## Abstract

In this paper, we consider optimal control problems (OCPs) applied to large-scale linear dynamical systems with a large number of states and inputs. We attempt to reduce such problems into a set of independent OCPs of lower dimensions. Our decomposition is ‘exact’ in the sense that it preserves all the information about the original system and the objective function. Previous work in this area has focused on strategies that exploit symmetries of the underlying system and of the objective function. Here, instead, we implement the algebraic method of simultaneous block diagonalization of matrices (SBD), which we show provides advantages both in terms of the dimension of the subproblems that are obtained and of the computation time. We provide practical examples with networked systems that demonstrate the benefits of applying the SBD decomposition over the decomposition method based on group symmetries.

## I. INTRODUCTION

Optimal control of high-dimensional dynamical systems and networks has recently attracted much attention from the scientific community, see e.g., [1], [2], [3], [4], [5], [6], [7], [8]. A major issue with computing the solution of optimal control problems with many states and inputs is the computational complexity of the matrix operations that must be performed. In addition, running these operations in parallel is often nontrivial. Here we propose a novel method to decouple large optimal control problems into independent subproblems of the lowest dimension, which also provides a way to parallelize the solution.

A vast literature [9], [10], [11] has focused on reducing the dimensionality of complex dynamical systems. The fundamental approach of model reduction is to generate a lower order model that preserves key features of the original system, see e.g., [12], [13], [14], [15], [16], [17]. Typically, this implies that information about the dominant modes/components of the system is preserved, while information about the non-dominant modes/components is removed. Our approach in this paper is to consider decompositions that are ‘exact’ in the sense that all the information about the original system and the original objective function is preserved.

Symmetries are common in both natural and engineering systems [18], [19], [20], [21], [22], [23]. The analysis of symmetries has been exploited in various disciplines such as semi-definite programming [24], [25], [26], network synchronization [27], [28], arithmetic optimization methods [29], the backward computation of reachable sets for nonlinear discrete-time control systems [30], and stability of interconnected subsystems [31], thus making it a popular approach for reducing the computation complexity associated with high dimensional problems.

Several studies have exploited symmetry to decouple linear OCPs involving large-scale systems into simpler subsystems. For example, Ref. [32] used symmetry to reduce the computational complexity involved in the controller synthesis of a linear system. Ref. [33] analyzed networked systems with symmetries in their interconnection topology and obtained a dimension reduction in their certification performance. Ref. [34] exploited symmetries in interior-point algorithms involved in the solution of optimization problems. References [35], [36], [37] used symmetry to reduce the computational complexity required by the solution of model predictive control problems. While all these papers have exploited symmetry to reduce the computational complexity involved in the solution of problems of different nature, in this paper we recognize that sometimes dimensionality reductions are possible even in systems that do not possess symmetry. As an example of this, the reader may consider the distinction between ‘orbit partitions’ and ‘equitable partitions’ in graph theory [38], where the former is generated by symmetry, while the latter is not. References [26], [32], [36], [39], [40], [41] have focused on a decomposition based on the symmetries of the system and, if applicable, of the objective function, which requires calculation of the irreducible representations (IRR) of the symmetry group. The goal of this paper is to compare the symmetry approach with an alternative symmetry-independent approach, which we will show yields better and faster decompositions.

In particular, we present a decomposition strategy, based on the techniques for simultaneous block diagonalization (SBD) of matrices, which is exact in the sense that it preserves all the available information about the system and the objective function (in contrast to model reduction methods) and requires fewer assumptions in comparison with the group theory approach. One example shows that our SBD approach can decompose an OCP into smaller subproblems than the symmetry-based decomposition. Furthermore, the decoupled OCPs resulting from the application of the SBD transformation have the finest size; hence, the SBD transformation is guaranteed to reduce the original problem into subproblems of the lowest dimension. Computing the decomposition with our method is also typically faster than with the symmetry approach.

In Section II, we introduce the preliminaries that are used throughout this paper. In Section III, we discuss the fundamentals of the SBD problem and briefly describe how to find the proper transformation matrix. In Section IV, we decouple the OCP for large-scale systems into a set of lower-dimensional OCPs. In Section V, we discuss how the SBD approach can be applied to a large-scale system in the form of a network of coupled sub-systems. In Section VI, we demonstrate the effectiveness of our method when compared to the symmetry-based approach by using three examples. Finally, the conclusions are presented in Section VII.

## II. PRELIMINARIES

Here, we introduce the mathematical notation that will be used throughout this paper. We denote with 𝓝(⋅) the null subspace of a matrix.

### Definition 1:

The vectorizing function vec : ${\mathbb{R}}^{n\times m}\mapsto {\mathbb{R}}^{nm}$ takes a matrix as input and returns a vector by stacking the columns of the matrix on top of each other.

**Algorithm 1:** The SBD Transformation [46].

**Input:** $A_{1},A_{2},\ldots ,A_{M}\in {\mathbb{R}}^{n\times n}$	
**Output:** *T*	
1:	$P_{i}=I_{n}⊗A_{i}-A_{i}^{⊤}⊗I_{n},\;i=1,\ldots ,M$
2:	$S=\sum_{i=1}^{M}P_{i}^{⊤}P_{i}$
3:	vec(U)=𝓝(S)
4:	Reshape vec(*U*) into $U\in {\mathbb{R}}^{n\times n}$ and set $U:=(1/2)\left(U+U^{⊤}\right)$
5:	*T* is a matrix with eigenvectors of *U* as its columns
6:	**return** *T*

Taken two matrices $A\in {\mathbb{R}}^{n\times m}$ and $B\in {\mathbb{R}}^{p\times q}$, the Kronecker product $A⊗B\in {\mathbb{R}}^{np\times mq}$ and the direct sum A⊕B∈ℝ(n+p)×(m+q) are defined as follows,

$$A⊗B=\left[\begin{array}{ccc} a_{11}B & \ldots & a_{1m}B\\ \vdots & \ddots & \vdots \\ a_{n1}B & \ldots & a_{nm}B \end{array}\right],\;A⊕B=\left[\begin{array}{cc} A & 𝟘\\ 𝟘 & B \end{array}\right].$$

An important property of the Kronecker product is the mixed-product property: taking two square matrices *C* and *D* with the same dimension *m*, and other two square matrices *E* and *F* with the same dimension *n*, the *nm*-dimensional square matrix (C⊗E)(D⊗F)=(CD⊗EF). A property of the direct sum is that if *G* and *H* are square and invertible matrices, then ${\left(G⊕H\right)}^{-1}=G^{-1}⊕H^{-1}$ [42].

## III. BACKGROUND

In this section, the SBD decomposition is introduced, and an algorithm to calculate the similarity transformation, which we will use throughout this paper, is provided.

### Definition 2.

*Simultaneous Block Diagonalization of Matrices (SBD) [43], [44], [45], [46]:* Given a set of *M* square *n*-dimensional matrices $𝓢=\left\{A_{1},A_{2},\ldots ,A_{M}\right\}$, an SBD transformation is an orthogonal square matrix *T* (herefater, transformation matrix) with dimension *n* such that

(1) $$T^{-1}A_{k}T={⊕}_{j=1}^{l}B_{j}^{k},\;k=1,2,\ldots ,M,$$

where the symbol ⊕ is the direct sum of matrices, *l* denotes the number of blocks, each block $B_{j}^{k}$ is a square matrix with the dimension *b*_*j*_ and $\sum_{j=1}^{l}b_{j}=n$.

### Definition 3.

*Finest SBD:* The finest SBD is an SBD for which the resulting blocks cannot be further refined by any other similarity transformation [43].

Maehara, Murota et al. proposed algebraic techniques to SBD a set of matrices in [44], [45], [46]. Their approach is to find a basis that diagonalizes the *-algebra associated with the algebra generated by 𝓢. Among other applications, the SBD approach has been used to decouple the stability analysis of networks of oscillators, see for example [47], [48], [49], [50]. We present below Algorithm 1 introduced in [46] to find the transformation *T*. In Section I of the Supplementary Information, we discuss the effects of the sparsity level of the input matrices on the computation time of the Algorithm 1.

The mathematical proofs that provide support to Algorithm 1 can be found in [46]. Other references [46], [47], [48] also provide MATLAB codes of other numerical SBD algorithms.

## IV. DECOUPLING OPTIMAL CONTROL PROBLEMS

Consider the OCP,

(2) $$\underset{u}{\min}\;\bar{J}=\frac{1}{2}\int_{0}^{t_{f}}\left(x^{⊤}Qx+{\bar{u}}^{⊤}\bar{R}\bar{u}+2x^{⊤}\bar{F}u\right)dt,\;\text{s.t.}\;\dot{x}(t)=Ax(t)+\bar{B}\bar{u}(t),x(0)=x_{0},x\left(t_{f}\right)=x_{f},$$

where $x\in {\mathbb{R}}^{n}$ is the vector of the system states, and $\bar{u}\in {\mathbb{R}}^{m}(n\geq m)$ is the vector of the system inputs, and $A\in {\mathbb{R}}^{n\times n}$, $\bar{B}\in {\mathbb{R}}^{n\times m}\cdot Q\in {\mathbb{R}}^{n\times n}$, $Q\underline{≻}0$, and $\bar{R}\in {\mathbb{R}}^{m\times m}$, $\bar{R}≻0.\bar{F}\in {\mathbb{R}}^{n\times m}$, where $Q-\bar{F}{\bar{R}}^{-1}{\bar{F}}^{⊤}\underline{≻}0$. Under the assumption that the pair (*A*, *B*) is controllable and that the pair $(A-\bar{B}{\bar{R}}^{-1}{\bar{F}}^{⊤},Q-\bar{F}{\bar{R}}^{-1}{\bar{F}}^{⊤})$ is observable, a unique solution for (2) exists. We rewrite (2) as

(3a) $$\underset{u}{\min}J=\frac{1}{2}\int_{0}^{t_{f}}\left(x^{⊤}Qx+u^{⊤}Ru+2x^{⊤}Fu\right)dt,$$

(3b) $$\text{s.t.}\;\dot{x}(t)=Ax(t)+Bu(t),\;x(0)=x_{0},\;x\left(t_{f}\right)=x_{f},$$

where now all the matrices $A,B,Q,R,F\in {\mathbb{R}}^{n\times n}$, and both vectors $x\in {\mathbb{R}}^{n}$, and $u\in {\mathbb{R}}^{n}$. In (3) we set,

(4) $$\begin{array}{l} u(t)=\left[\begin{array}{c} \bar{u}(t)\\ u_{O}(t) \end{array}\right],B=\left[\begin{array}{cc} \bar{B} & {𝟘}_{n\times (n-m)} \end{array}\right],R=\bar{R}⊕R_{O},\\ \;F=\left[\begin{array}{cc} \bar{F} & {𝟘}_{n\times (n-m)} \end{array}\right]; \end{array}$$

where the additional input vector $u_{O}(t)\in {\mathbb{R}}^{n-m}$ is appended to $\bar{u}(t)$ so that $u\in {\mathbb{R}}^{n}$, and $R_{O}\in {\mathbb{R}}^{\left(n-m)\times (n-m\right)}$ is an arbitrary positive definite matrix. The additional inputs do not affect the dynamics since their corresponding coefficients in *B* and *F* are zero.

### Remark 1:

The solution ${\bar{u}}^{*}(t)$ of the OCP with (2) coincides with the solution ${\bar{u}}^{*}(t)$ of the OCP with (3), with $u_{O}^{*}(t)=0$.

This follows from the fact that the particular choice of *R*_*O*_ and *u*_*O*_ is independent of the original OCP. The modified OCP will break into two independent OCPs, one in $\bar{u}$, and one in $u_{O}$.

The orthogonal similarity transformation matrix *T*,

(5) T=SBD(A,B,Q,R,F),

is applied to decouple the original large problem into a set of *L* lower dimensional problems by simultaneously block diagonalizing the following matrices,

(6a) $$\tilde{A}=\overset{L}{\underset{l=1}{⊕}}{\tilde{A}}_{l}=T^{⊤}AT,\;\tilde{B}=\overset{L}{\underset{l=1}{⊕}}{\tilde{B}}_{l}=T^{⊤}BT,$$

(6b) $$\tilde{Q}=\overset{L}{\underset{l=1}{⊕}}{\tilde{Q}}_{l}=T^{⊤}QT,\;\tilde{R}=\overset{L}{\underset{l=1}{⊕}}{\tilde{R}}_{l}=T^{⊤}RT,$$

(6c) $$\tilde{F}=\overset{l=1}{\underset{L}{⊕}}{\tilde{F}}_{l}=T^{⊤}FT.$$

The blocks ${\tilde{A}}_{l},{\tilde{B}}_{l},{\tilde{Q}}_{l},{\tilde{R}}_{l},{\tilde{F}}_{l}$ all have the same size $\sum_{l=1}^{L}n_{l}=n$. (3b) is pre-multiplied by $T^{⊤}$,

(7) $$T^{⊤}\dot{x}(t)=T^{⊤}ATT^{⊤}x(t)+T^{⊤}BTT^{⊤}u(t).$$

By defining $z(t):=T^{⊤}x(t)$ and $v(t):=T^{⊤}u(t)$, and using results from (6) and (7), (3b) is rewritten,

(8) $$\dot{z}(t)=\tilde{A}z(t)+\tilde{B}v(t),\;z(0)=z^{0}=T^{⊤}x_{0}\;z\left(t_{f}\right)=z^{f}=T^{⊤}x_{f},$$

The cost *J* from (3a) is rewritten in terms of the new variables by setting x(t)=Tz(t) and u(t)=Tv(t) as

$$J=\frac{1}{2}\int_{0}^{t_{f}}\left(z^{⊤}T^{⊤}QTz+v^{⊤}T^{⊤}RTv+2z^{⊤}T^{⊤}FTv\right)dt\;\;=\frac{1}{2}\int_{0}^{t_{f}}\left(z^{⊤}\tilde{Q}z+v^{⊤}\tilde{R}v+2z^{⊤}\tilde{F}v\right)dt.$$

So, the transformed OCP is

(9a) $$\underset{z}{\min}J=\frac{1}{2}\int_{0}^{t_{f}}\left(z^{⊤}\tilde{Q}z+v^{⊤}\tilde{R}v+2z^{⊤}\tilde{F}v\right)dt,$$

(9b) $$\text{s.t.}\;\dot{z}(t)=\tilde{A}z(t)+\tilde{B}v(t),z(0)=z^{0},\;z\left(t_{f}\right)=z^{f}.$$

### Lemma 1:

The large dimensional optimization problem from (9) decouples into a set of smaller independent sub-problems,

(10) $$\underset{v_{l}}{\min}\;J_{l}=\frac{1}{2}\int_{0}^{t_{f}}\left(z_{l}^{⊤}{\tilde{Q}}_{l}z_{l}+v_{l}^{⊤}{\tilde{R}}_{l}v_{l}+2z_{l}^{⊤}{\tilde{F}}_{l}v_{l}\right)dt,\;\;\text{s.t.}\;{\dot{z}}_{l}(t)={\tilde{A}}_{l}z_{l}(t)+{\tilde{B}}_{l}v_{l}(t),\;z_{l}(0)=z_{l}^{0},\;z_{l}\left(t_{f}\right)=z_{l}^{f},$$

l=1,…,L, with $z(t)={\left[z_{1}{\left(t\right)}^{⊤}\ldots z_{L}{\left(t\right)}^{⊤}\right]}^{⊤}$, $v(t)={\left[v_{1}{\left(t\right)}^{⊤}\ldots v_{L}{\left(t\right)}^{⊤}\right]}^{⊤}$, and $J=\sum_{l=1}^{L}J_{l}$.

#### Proof:

After applying the transformation *T*, the OCP from (3) is transformed to,

$$\underset{v_{1},\ldots ,v_{L}}{\min}\;J_{1}+\cdots +J_{L}\;\text{s.t.}\;{\dot{z}}_{l}(t)={\tilde{A}}_{l}z_{l}(t)+{\tilde{B}}_{l}v_{l}(t),\;\;z_{l}(0)=z_{l}^{0},\;z_{l}\left(t_{f}\right)=z_{l}^{f},\;l=1,\ldots ,L,$$

where $J_{l}=\frac{1}{2}\int_{0}^{t_{f}}\left(z_{l}^{⊤}{\tilde{Q}}_{l}z_{l}+v_{l}^{⊤}{\tilde{R}}_{l}v_{l}+2z_{l}^{⊤}{\tilde{F}}_{l}v_{l}\right)dt$, and $\forall i,j,i\neq j,J_{i}$ and $J_{j}$ are independent from one another and $J_{i}=J_{i}\left(z_{i},v_{i}\right)\geq 0$, and $J_{j}=J_{j}\left(z_{j},v_{j}\right)\geq 0$. Also, the states $z_{i},z_{j}$ and the inputs $v_{i},v_{j}$ are decoupled, so we conclude the large OCP is decoupled into *L* independent OCPs. This concludes the proof.

### Remark 2:

The dimension of the controllable and observable subspaces and the open-loop poles of the transformed system (8) are the same as those of the system (3).

This is due to the fact that we have obtained the transformed system by application of a similarity transformation, which preserves the rank and the eigenvalues of the matrices [51].

We have provided an algebraic method to decouple a large OCP into a set of *L* smaller OCPs. There are two advantages of this decomposition. The first one is that the lower-dimension OCPs are easier to solve since the algebraic operations have lower computational complexity for lower-dimension matrices; the second one is that the lower-dimension OCPs can be solved in parallel (since they are independent of one another.)

Next, we discuss in detail how the closed-form solutions of the sample OCP (3) is affected by the SBD transformation. The Hamiltonian for the OCP (3) is,

(11) $$ℋ=\frac{1}{2}\left(x^{⊤}Qx+u^{⊤}Ru+2x^{⊤}Fu\right)+\lambda^{⊤}(Ax+Bu),$$

where $\lambda (t)\in {\mathbb{R}}^{n}$ is the time-varying costate vector. By solving the following equations simultaneously,

(12a) $$\dot{x}(t)=\frac{\partial ℋ}{\partial \lambda (t)}=Ax(t)+Bu(t),$$

(12b) $$-\dot{\lambda }(t)=\frac{\partial ℋ}{\partial x(t)}=Qx(t)+Fu(t)+A^{⊤}\lambda (t),$$

(12c) $$0=\frac{\partial ℋ}{\partial u(t)}=F^{⊤}x(t)+Ru(t)+B^{⊤}\lambda (t).$$

the optimal solution can be found. From (12c), we obtain:

(13) $$u^{*}(t)=-R^{-1}\left(B^{⊤}\lambda^{*}(t)+F^{⊤}x^{*}(t)\right).$$

Now, (12a) and (12b) should be solved for x(t) and λ(t). By assuming λ(t)=Px(t)+ξ(t), we write,

(14a) $$\left[\begin{array}{c} \dot{x}(t)\\ \dot{\xi }(t) \end{array}\right]=\left[\begin{array}{cc} \hat{A} & \hat{B}\\ \hat{Q} & -{\hat{A}}^{⊤} \end{array}\right]\left[\begin{array}{c} x(t)\\ \xi (t) \end{array}\right],$$

(14b) $$\hat{A}=A-BR^{-1}\left(F^{⊤}+B^{⊤}P\right),$$

(14c) $$\hat{B}=-BR^{-1}B^{⊤},$$

(14d) $$\begin{array}{c} \hat{Q}=P\left(A-BR^{-1}F^{⊤}\right)+\left(A^{⊤}-FR^{-1}B^{⊤}\right)P\\ -PBR^{-1}B^{⊤}P+Q-FR^{-1}F^{⊤}. \end{array}$$

To decouple x and ξ, we find *P* that sets $\hat{Q}=𝟘$. Note that $\hat{Q}=𝟘$ is the standard form of the algebraic Riccati equation in the matrix *P* [52],

(15) $$\begin{array}{c} P\left(A-BR^{-1}F^{⊤}\right)+\left(A^{⊤}-FR^{-1}B^{⊤}\right)P\\ -PBR^{-1}B^{⊤}P+Q-FR^{-1}F^{⊤}=𝟘. \end{array}$$

After solving for *P*, (14a) can be solved for ξ and x independently. To find the analytical solution, we set $\xi (t)=e^{{\hat{A}}^{⊤}\left(t_{f}-t\right)}\xi_{f}$ where $\xi_{f}={\hat{W}}^{-1}\left(x_{f}-e^{\hat{A}t_{f}}x_{0}\right)$ and,

(16) $$\hat{W}=\int_{0}^{t_{f}}e^{\hat{A}\left(t_{f}-\tau \right)}\hat{B}e^{{\hat{A}}^{⊤}\left(t_{f}-\tau \right)}d\tau .$$

Finding $u^{*}(t)$ is now straightforward: by solving (15) for *P*, and then solving (14a) for x(t) and ξ(t) to evaluate λ(t), we can be substitute back in (13) to find $u^{*}(t)$.

In general, solving (15) and (16) can be computationally challenging for large *n*. Using the SBD decomposition, the *n* × *n* algebraic Riccati equation from (15) and the *n* × *n* controllability Gramian from (16) are broken into *L* smaller independent equations in the blocks of the matrices $\tilde{P}={⊕}_{l=1}^{L}{\tilde{P}}_{l}$, and $\tilde{W}={⊕}_{l=1}^{L}{\tilde{W}}_{l}$,

(17) $$\begin{array}{c} {\tilde{P}}_{l}\left({\tilde{A}}_{l}-{\tilde{B}}_{l}{\tilde{R}}_{l}^{-1}{\tilde{F}}_{l}^{⊤}\right)+\left({\tilde{A}}_{l}^{⊤}-{\tilde{F}}_{l}{\tilde{R}}_{l}^{-1}{\tilde{B}}_{l}^{⊤}\right){\tilde{P}}_{l}\\ -{\tilde{P}}_{l}{\tilde{B}}_{l}{\tilde{R}}_{l}^{-1}{\tilde{B}}_{l}^{⊤}{\tilde{P}}_{l}+{\tilde{Q}}_{l}-{\tilde{F}}_{l}{\tilde{R}}_{l}^{-1}{\tilde{F}}_{l}^{⊤}=𝟘, \end{array}$$

(18) $${\tilde{W}}_{l}=\int_{0}^{t_{f}}e^{{\hat{A}}_{l}\left(t_{f}-\tau \right)}{\hat{B}}_{l}e^{{\hat{A}}_{l}^{⊤}\left(t_{f}-\tau \right)}d\tau .$$

The computational complexities associated with solving the Riccati equation and the controllability Gramian are ${𝓞}_{R}\left(n^{3}\right)$ [53] and ${𝓞}_{G}\left(n^{4}\right)$ [54], respectively. By means of the decomposition, the overall complexity reduces to $\sum_{l=1}^{L}{𝓞}_{R}\left({\left(n_{l}\right)}^{3}\right)\leq {𝓞}_{R}\left(n^{3}\right)$, and $\sum_{l=1}^{L}{𝓞}_{G}\left({\left(n_{l}\right)}^{4}\right)\leq {𝓞}_{G}\left(n^{4}\right)$, where the equality corresponds to the case *L* = 1.

### Remark 3:

The solution of (17) can be transformed back to obtain the solution of (15), i.e., $P=T\tilde{P}T^{⊤}$.

To show this, consider (17) and construct $\tilde{P}$,

(19) $$\begin{array}{l} \tilde{P}\left(\tilde{A}-\tilde{B}{\tilde{R}}^{-1}{\tilde{F}}^{⊤}\right)+\left({\tilde{A}}^{⊤}-\tilde{F}{\tilde{R}}^{-1}{\tilde{B}}^{⊤}\right)\tilde{P}\\ \;-\tilde{P}\tilde{B}{\tilde{R}}^{-1}{\tilde{B}}^{⊤}\tilde{P}+\tilde{Q}-\tilde{F}{\tilde{R}}^{-1}{\tilde{F}}^{⊤}=𝟘, \end{array}$$

(20) $$\begin{array}{l} \tilde{P}\left(T^{⊤}AT-T^{⊤}BTT^{⊤}R^{-1}TT^{⊤}F^{⊤}T\right)\\ \;+\left(T^{⊤}A^{⊤}T-T^{⊤}FTT^{⊤}R^{-1}TT^{⊤}B^{⊤}T\right)\tilde{P}\\ \;-\tilde{P}T^{⊤}BTT^{⊤}R^{-1}TT^{⊤}B^{⊤}T\tilde{P}+T^{⊤}QT\\ \;-T^{⊤}FTT^{⊤}R^{-1}TT^{⊤}F^{⊤}T=𝟘, \end{array}$$

(21) $$\begin{array}{l} \tilde{P}T^{⊤}\left(A-BR^{-1}F^{⊤}\right)T+T^{⊤}\left(A^{⊤}-FR^{-1}B^{⊤}\right)T\tilde{P}\\ \;-\tilde{P}T^{⊤}BR^{-1}B^{⊤}T\tilde{P}+T^{⊤}QT-T^{⊤}FR^{-1}F^{⊤}T=𝟘, \end{array}$$

After pre-multiplying and post-multiplying (21) by *T* and $T^{⊤}$, respectively, we get,

(22) $$\begin{array}{l} \left(T\tilde{P}T^{⊤}\right)\left(A-BR^{-1}F^{⊤}\right)+\left(A^{⊤}-FR^{-1}B^{⊤}\right)\left(T\tilde{P}T^{⊤}\right)\\ \;-\left(T\tilde{P}T^{⊤}\right)BR^{-1}B^{⊤}\left(T\tilde{P}T^{⊤}\right)+Q-FR^{-1}F^{⊤}=𝟘. \end{array}$$

Hence, $P=T\tilde{P}T^{⊤}$ is the transformed solution of the original Riccati equation. The controllability Gramian also reduces to smaller-dimension Gramians after the application of the transformation *T*. Similar derivation for $\tilde{W}$ will show that $\hat{W}=T\tilde{W}T^{⊤}$.

### Remark 4:

We note here that the case of the infinite horizon OCP can be decomposed in a similar fashion, see Section II of the Supplementary Information.

To conclude, the large OCP in (3) has been decoupled into a set of smaller OCPs in (10) by the simultaneous application of the orthogonal similarity transformation matrix *T* to the system dynamics and the quadratic cost. By solving the lower dimensional OCPs separately, the control law for the large system is then recovered.

## V. DECOUPLING NETWORKED SYSTEMS

Networked systems are formed of individual systems coupled together to form a network. Many real-world systems are naturally described as networked systems. For instance, social networks, power grids, and computer networks are all examples of networked systems. A large literature has dealt with optimal control of networked systems [55], [56], [57], [58], [59], [60], [61], [62], [63].

In this paper, we consider a networked system defined by the graph 𝓖(𝓥,ℰ), where 𝓥={i=1,…,N} is the set of *N* nodes/vertices and ℰ⊆𝓥×𝓥 is the set of edges, i.e., ordered pairs of nodes (i,j)∈𝓥×𝓥. Each node of the graph is a subsystem and an edge between two nodes represents coupling between them. We also assume that the graph 𝓖 is heterogeneous, i.e, it contains different types of nodes. Thus, the set of nodes 𝓥 can be partitioned into *S* clusters formed of nodes of the same type, ${𝓒}_{1},{𝓒}_{2},\ldots ,{𝓒}_{S}$, $\cup_{s=1}^{S}{𝓒}_{s}=𝓥$, ${𝓒}_{s}\cap {𝓒}_{l}=\phi$ for s≠l, with $N=\sum_{s=1}^{S}N_{s}$ where *N*_*s*_ is the number of nodes in cluster ${𝓒}_{s}$, S≤N. The dynamics of this networked system consisting of *N n*-dimensional interacting sub-systems obeys [64],

(23) $${\dot{x}}_{i}(t)=A_{i*}x_{i}(t)+B_{i*}u_{i}(t)+\sum_{j=1}^{N}G_{ij}H_{j*}x_{j}(t),\;\;i*=s\;\text{if}\;i\in {𝓒}_{s}$$

i=1,…,N and i∗=1,…,S. The subscript i∗ labels the unique cluster ${𝓒}_{i*}$ to which node *i* belongs. Hence, i∗ also identifies the dynamical matrices $A_{i*}$, $B_{i*}$, and $H_{i*}$ for the sub-system *i*. The term $A_{i*}x_{i}(t)$, $A_{i*}\in {\mathbb{R}}^{n\times n}$ describes the dynamics of each sub-system, the term $B_{i*}u_{i}(t)$, $B_{i*}\in {\mathbb{R}}^{n\times m}$ describes the effect of the control signals on each sub-system, and $\sum_{j=1}^{N}G_{ij}H_{j*}x_{j}(t)$, $G\in {\mathbb{R}}^{N\times N}$ and $H_{j*}\in {\mathbb{R}}^{n\times n}$ is the overall coupling that sub-system *i* receives from the other sub-systems in the network. The symmetric matrix $G=\left[G_{ij}\right]$ is the adjacency matrix that describes how the individual network systems are coupled to one another, namely if systems *i* and *j* are coupled, $G_{ij}=G_{ji}\neq 0$, otherwise $G_{ij}=G_{ji}=0$.

### Definition 4:

We define the *N*-dimensional indicator matrix *E*_*s*_ as,

(24) $$E_{s}=e_{ij}=\{\begin{array}{ll} 1 & \text{if}\;i=j\;\text{\&}\;i\in {𝓒}_{s}\\ 0 & \text{otherwise} \end{array}.$$

Given a general networked system represented by the graph 𝓖, and system dynamics (23), one can follow the 4 steps below to decouple the system into multiple OCPs and solve them in parallel to reduce the computation time.

### 1) Obtain the State Space Representation:

The first step is to convert the dynamics of the system to a standard state space form. We do so by first reordering the system states. Without loss of generality, the first *N*_1_ sub-systems are set to be of type 1, the second *N*_2_ sub-systems to be of type 2, etc. This results in the following indicator matrices, *E*_*s*_ for s=1,…,S

(25) $$E_{1}=\left[\begin{array}{cc} I_{N_{1}} & 𝟘\\ 𝟘 & {𝟘}_{N-N_{1}} \end{array}\right],E_{2}=\left[\begin{array}{ccc} {𝟘}_{N_{1}} & 𝟘 & 𝟘\\ 𝟘 & I_{N_{2}} & 𝟘\\ 𝟘 & 𝟘 & {𝟘}_{N-\left(N_{1}+N_{2}\right)} \end{array}\right],\;\;\ldots ,E_{S}=\left[\begin{array}{cc} {𝟘}_{N-N_{S}} & 𝟘\\ 𝟘 & I_{N_{S}} \end{array}\right].$$

The new re-ordered system can now be easily converted to the desired form. Using (25), (23) can be rewritten in the following compact form,

(26) $$\begin{array}{c} \dot{X}(t)=\left[\sum_{s=1}^{S}\left(E_{s}⊗A_{s}+GE_{s}⊗H_{s}\right)\right]X(t)\\ +\left[\sum_{s=1}^{S}E_{s}⊗B_{s}\right]U(t), \end{array}$$

where $X(t)={\left[x_{1}{\left(t\right)}^{⊤}\ldots x_{N}{\left(t\right)}^{⊤}\right]}^{⊤}\in {\mathbb{R}}^{Nn}$, $U(t)={\left[u_{1}{\left(t\right)}^{⊤}\ldots u_{N}{\left(t\right)}^{⊤}\right]}^{⊤}\in {\mathbb{R}}^{Nm}$.

It can be seen that (26) is a standard state space representation with the vector ***X*** representing the states and the vector ***U*** representing the inputs.

### 2) Define the Cost Function:

The second step is to define a cost function depending on the particular OCP. Given the system is in the standard state space representation, a quadratic cost function can be written as,

(27) $$J=\int_{0}^{t_{f}}\left(X^{⊤}\left(Q⊗W_{Q}\right)X+U^{⊤}\left(R⊗W_{R}\right)U\right)dt.$$

The matrices *Q* and *W*_*Q*_ are positive semi-definite (and so is the matrix $Q⊗W_{Q}$). The matrices *R* and *W*_*R*_ are positive definite (and so is the matrix $R⊗W_{R}$). In particular, $Q\in {\mathbb{R}}^{N\times N}$ and $R\in {\mathbb{R}}^{N\times N}$ are weighing matrices for the network systems. These matrices contain information on the weights given to the states and inputs of each sub-system relative to the other sub-systems. Instead, the matrices $W_{Q}\in {\mathbb{R}}^{n\times n}$ and $W_{R}\in {\mathbb{R}}^{m\times m}$ are weighing matrices for the individual sub-system states and inputs respectively. These matrices measure how the states (inputs) of a single sub-system are weighted relative to other states (inputs) of the same sub-system. For example, by properly choosing these matrices, one can drive individual sub-system states towards desirable values while limiting the effort to do so.

### 3) Add Additional Input Channels if needed:

This is done in an analogous way to the process to obtain (3) from (2) described in Section IV. Namely, a sub-system with *m* number of input channels, *m* < *n* needs to be transformed to a subsystem with *n* number of input channels. This can be done by adding *n* – *m* input channels; hence $u_{i}\in {\mathbb{R}}^{n}$ and $U\in {\mathbb{R}}^{Nn}$, and we set their corresponding coefficients to zero in the input matrix, $B_{i*}$. Doing so does not change the original dynamics of the system. Further, we replace the matrix *W*_*R*_ by a new matrix $W_{R}^{\text{new}}=W_{R}^{\text{old}}⊕W_{O}$ where $W_{O}\in {\mathbb{R}}^{\left(n-m)\times (n-m\right)}$ is an arbitrary positive definite matrix.

### 4) Apply the SBD Transformation:

The final step is to apply the SBD transformation. In order to show how the system dynamics and the objective function are decoupled by our proposed method, we present some key properties of the SBD transformation matrix. In particular, we define the concept of a canonical SBD transformation.

### Definition 5:

The transformation matrix *T* is a *canonical SBD transformation* if each indicator matrix, $E_{S},s=1,\ldots ,S$, maps back to itself (but with permuted rows and columns) after the application of the transformation *T*. A canonical transformation matrix is block-diagonal in *S* blocks, and the size of each block is equal to $N_{1},N_{2},\ldots ,N_{S}$.

This follows from the property that in order to commute with $E_{1},E_{2},\ldots ,E_{S}$, the matrix *U* from Algorithm 1 must have the same block diagonal structure as $E_{S},s=1,\ldots ,S$. Hence, since the columns of the matrix *T* are the eigenvectors of *U*, *T* must have the same block structure as *U*. Moreover, because of the block structure of both the matrix *T* and the matrices $E_{S},s=1,\ldots ,S$, we have that $T^{T}E_{S}T$ maps back to *E*_*s*_, but possibly with permuted rows and columns.

Next, we consider the application of a canonical SBD transformation matrix *T* to the optimal control problem (26). The matrix

(28) $$T=SBD\left(E_{1},\ldots ,E_{S},\;G,\;Q,\;R\right)$$

transforms the matrices $E_{1},\ldots ,E_{S}$, *G*, *Q*, *R* into a block diagonal form with *P* blocks of dimension $n_{p},p=1,\ldots ,P$, $\sum_{p=1}^{P}n_{p}=N$ and *G*, *Q* and *R* into $\tilde{G}$, $\tilde{Q}$ and $\tilde{R}$ respectively.

(29) $$M_{s}=\overset{P}{\underset{p=1}{⊕}}M_{s}^{p}=T^{⊤}E_{S}T,\;s=1,\ldots ,S,$$

(30) $$\tilde{G}=\overset{P}{\underset{p=1}{⊕}}{\tilde{G}}^{p}=T^{⊤}GT,\;\tilde{Q}=\overset{P}{\underset{p=1}{⊕}}{\tilde{Q}}^{p}=T^{⊤}QT,$$

(31) $$\tilde{R}=\overset{P}{\underset{p=1}{⊕}}{\tilde{R}}^{p}=T^{⊤}RT.$$

where the *M*_*s*_ matrices are the transformed indicator matrices.

Note that each one of the matrices *M*_*s*_ is a diagonal matrix where the elements on the main diagonal are the same as those of the matrices *E*_*s*_ but permuted and consequently have the same block-diagonal structure as $\tilde{G}=T^{⊤}GT$, $\tilde{Q}=T^{⊤}QT$, and $\tilde{R}=T^{⊤}RT$. We now pre-multiply (26) by $T^{⊤}⊗I_{n}$, and define the following:

(32) $$z(t)=\left(T^{⊤}⊗I_{n}\right)X(t)\;v(t)=\left(T^{⊤}⊗I_{n}\right)U(t).$$

Thus, (26) can be re-written,

(33) $$\dot{z}(t)=\left(\sum_{s=1}^{S}M_{s}⊗A_{s}+\tilde{G}M_{s}⊗H_{S}\right)z(t)\;\;+\left(\sum_{s=1}^{S}M_{S}⊗B_{S}\right)v(t).$$

From (32), it is clear that $X(t)=\left(T⊗I_{n}\right)z(t)$ and $U(t)=\left(T⊗I_{n}\right)v(t)$. Therefore, the cost function from (27) is rewritten as,

(34) $$J=\int_{0}^{t_{f}}\left[z^{⊤}\left(\tilde{Q}⊗W_{Q}\right)z+v^{⊤}\left(\tilde{R}⊗W_{R}\right)v\right]dt.$$

Since the states and inputs are of the form, $z(t)={\left[z^{1}{\left(t\right)}^{⊤}\ldots z^{P}{\left(t\right)}^{⊤}\right]}^{⊤}$, $v(t)={\left[v^{1}{\left(t\right)}^{⊤}\ldots v^{P}{\left(t\right)}^{⊤}\right]}^{⊤}$, it is clear that the system dynamics has been decoupled into *P* independent equations and the cost function into *P* independent cost functions,

(35a) $$\begin{array}{c} {\dot{z}}^{p}(t)=\left(\sum_{s=1}^{S}M_{s}^{p}⊗A_{s}+{\tilde{G}}^{p}M_{s}^{p}⊗H_{s}\right)z^{p}(t)\\ +\left(\sum_{s=1}^{S}M_{s}^{p}⊗B_{S}\right)v^{p}(t), \end{array}$$

(35b) $$J^{p}=\int_{0}^{t_{f}}\left[z^{p⊤}\left({\tilde{Q}}^{p}⊗W_{Q}\right)z^{p}+v^{p⊤}\left({\tilde{R}}^{p}⊗W_{R}\right)v^{p}\right]dt,$$

where p=1,…,P, and $J=\sum_{p=1}^{P}J^{p}$.

Each OCP described by (35) can be solved independently and in parallel. Furthermore, the solution to the original OCP can be recovered by inverting the SBD transformation.

## VI. CASE STUDIES

The computation time for solving OCPs using our proposed approach depends on two major factors; 1) the dimensions of the OCPs resulting from the decomposition and 2) the time to compute the transformation matrix. The two examples that follow show that our proposed method based on the SBD transformation outperforms methods based on symmetry in both these aspects. A third example of HVAC control is used to demonstrate the effectiveness of our method.

### A. EXAMPLE 1: MASS-SPRING-DAMPER SYSTEM

**FIGURE 1.**
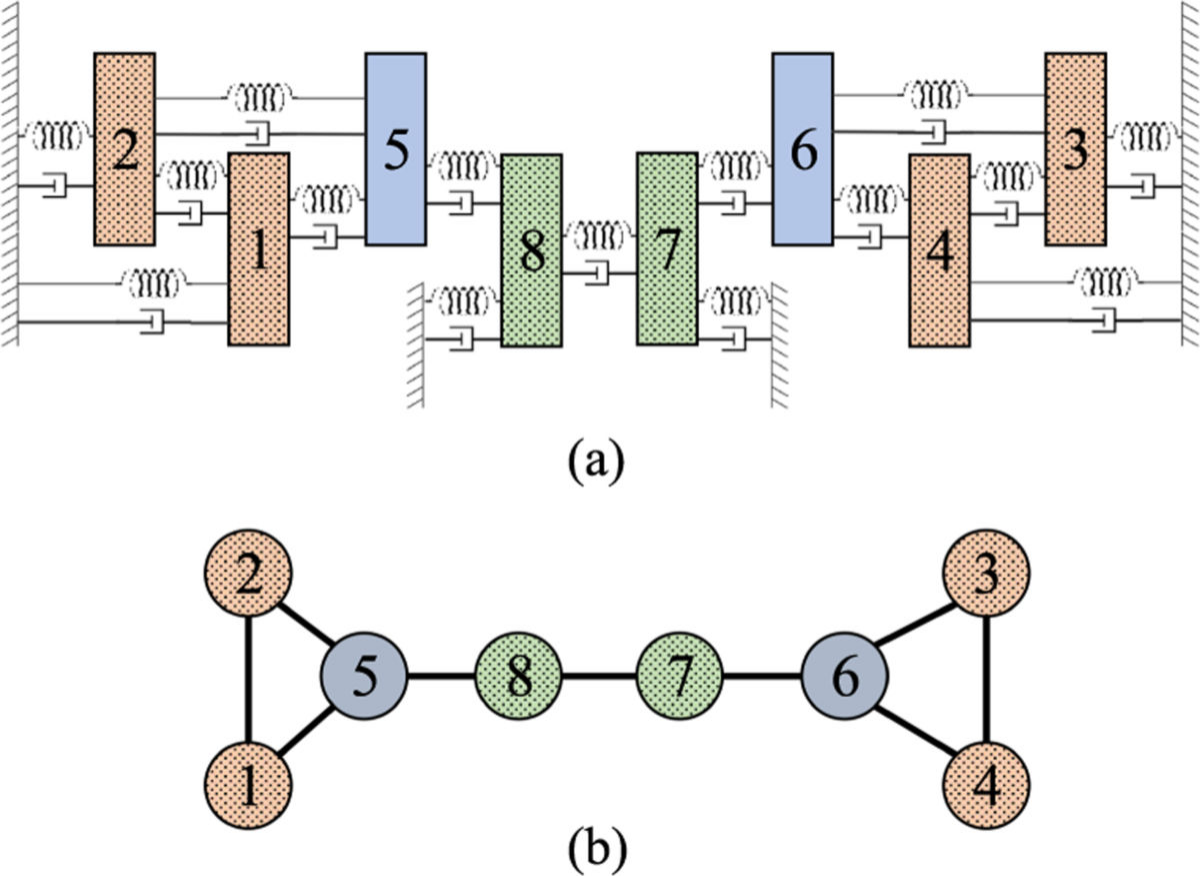
(a) Eight identical masses (*M*) are connected with identical linear springs (*K*) and dampers (*C*). Friction and gravity are negligible. $p_{1},\ldots ,p_{8}$ and $v_{1},\ldots ,v_{8}$ are positions and velocities of the masses. $u_{1},\ldots ,u_{8}$ are input forces. The direction of positive position, velocity, and input force is to the right-hand side of the figure. Masses of the same color are in the same orbital partition, and masses with the same background (either empty or dotted) are in the same equitable partition. (b) shows the topology of the connectivity between the masses. Here each node represents a mass and each edge represents a connection (spring and damper) between the connected masses.

With this example we show that the reduction based on our method produces finer blocks than the symmetry method. We compare the reduction from the SBD transformation with that of an IRR transformation applied to a system of connected mass-spring-dampers. Fig. 1 shows the topology of the connections between the masses. Here each node represents a mass and each edge represents a connection (spring and damper) between the connected masses.

Following the steps outlined in Section V, we first derive the state space representation of the system. Since all our masses are the same, *S* = 1, so we set $E_{S}=I_{8}$, $A_{S}=A$, $H_{s}=H$, and $B_{s}=B$,

(36) $$A=\left[\begin{array}{cc} 0 & 1\\ \frac{-3K}{M} & \frac{-3C}{M} \end{array}\right],\;H=\left[\begin{array}{cc} 0 & 0\\ \frac{K}{M} & \frac{C}{M} \end{array}\right],\;B=\left[\begin{array}{cc} 0 & 0\\ 1 & 0 \end{array}\right].$$

Note that the second column of the matrix B with entries that are all zeros is used here to make the dimensions of the matrices in (36) consistent. The adjacency matrix corresponding to the graph in Fig. 1 is,

(37) $$G=\left[\begin{array}{cccccccc} 0 & 1 & 0 & 0 & 1 & 0 & 0 & 0\\ 1 & 0 & 0 & 0 & 1 & 0 & 0 & 0\\ 0 & 0 & 0 & 1 & 0 & 1 & 0 & 0\\ 0 & 0 & 1 & 0 & 0 & 1 & 0 & 0\\ 1 & 1 & 0 & 0 & 0 & 0 & 0 & 1\\ 0 & 0 & 1 & 1 & 0 & 0 & 1 & 0\\ 0 & 0 & 0 & 0 & 0 & 1 & 0 & 1\\ 0 & 0 & 0 & 0 & 1 & 0 & 1 & 0 \end{array}\right].$$

The compact dynamical equation of the coupled masses via springs and dampers is

(38) $$\dot{X}(t)=\left[\left(I_{8}⊗A+G⊗H\right)\right]X(t)+\left[I_{8}⊗B\right]U(t).$$

For step 2, we consider a quadratic cost function as in (27). We set *R* = *I*_8_ and,

(39) $$Q=\left[\begin{array}{cccccccc} 1 & -1 & 0 & 0 & 0 & 0 & 0 & 0\\ -1 & 1 & 0 & 0 & 0 & 0 & 0 & 0\\ 0 & 0 & 1 & -1 & 0 & 0 & 0 & 0\\ 0 & 0 & -1 & 1 & 0 & 0 & 0 & 0\\ 0 & 0 & 0 & 0 & 2 & 0 & 0 & 0\\ 0 & 0 & 0 & 0 & 0 & 2 & 0 & 0\\ 0 & 0 & 0 & 0 & 0 & 0 & 1 & -1\\ 0 & 0 & 0 & 0 & 0 & 0 & -1 & 1 \end{array}\right].$$

This choice of *Q* indicates that we want to force masses 5 and 6 to go to the origin, while we want masses 1 and 2, masses 3 and 4, and masses 7 and 8 to follow the same trajectory (i.e., we want the difference between their positions and velocities to go to zero.) We note that the matrices *W*_*Q*_ and *W*_*R*_ do not play a role in finding the transformation *T*, but they are required to find the optimal control input $U^{*}(t)$.

Next, we compare our approach with the symmetry based approach. In order to compute the transformation matrix *P* that transforms the system in the irreducible representation coordinates, we use the algorithm proposed in [27]. A Python code of this algorithm is also provided in [65]. The orbital partition for this network is {1, 2, 3, 4}, {5, 6}, {7, 8}. The symmetry-based transformation

(40) $$P=\left[\begin{array}{cccccccc} 0 & 0 & -0.5 & 0 & 0 & -0.5 & \frac{-1}{\sqrt{2}} & 0\\ 0 & 0 & -0.5 & 0 & 0 & -0.5 & \frac{1}{\sqrt{2}} & 0\\ 0 & 0 & -0.5 & 0 & 0 & 0.5 & 0 & \frac{-1}{\sqrt{2}}\\ 0 & 0 & -0.5 & 0 & 0 & 0.5 & 0 & \frac{1}{\sqrt{2}}\\ 0 & \frac{-1}{\sqrt{2}} & 0 & 0 & \frac{-1}{\sqrt{2}} & 0 & 0 & 0\\ 0 & \frac{-1}{\sqrt{2}} & 0 & 0 & \frac{1}{\sqrt{2}} & 0 & 0 & 0\\ \frac{-1}{\sqrt{2}} & 0 & 0 & \frac{-1}{\sqrt{2}} & 0 & 0 & 0 & 0\\ \frac{-1}{\sqrt{2}} & 0 & 0 & \frac{1}{\sqrt{2}} & 0 & 0 & 0 & 0 \end{array}\right],$$

transforms *G* and *Q* into $\hat{G}=P^{⊤}GP$ and $\hat{Q}=P^{⊤}QP$,

(41a)

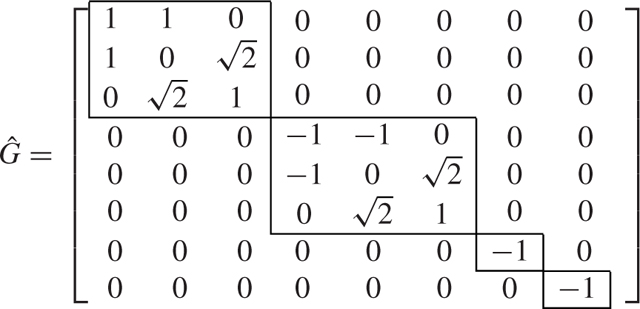



(41b)

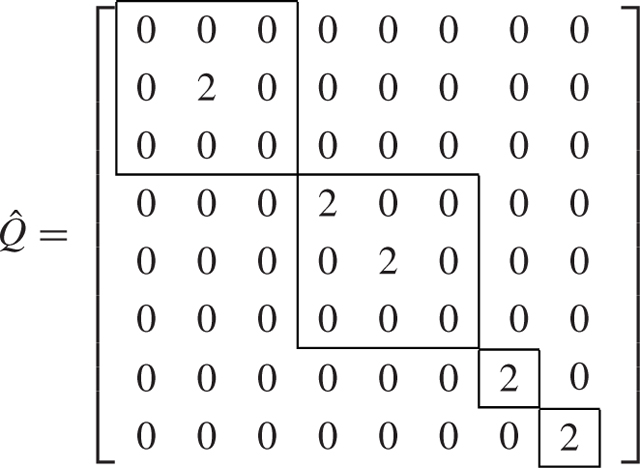


We see that the original *N* = 8-dimensional problem is decoupled into two 3-dimensional problems and two 1-dimensional problems by using the symmetry-based decomposition.

We now consider the SBD approach, and using the algorithm presented in [46], we compute the transformation matrix T=SBD(G,Q) which is equal to (42) shown at the bottom of the next page, and transforms the matrices *G* and *Q* into $\tilde{G}=T^{⊤}GT$ and $\tilde{Q}=T^{⊤}QT$.


(42) $$T=\left[\begin{array}{cccccccc} -0.15 & -0.20 & -0.43 & -0.27 & 0.29 & -0.65 & -0.23 & 0.34\\ -0.15 & -0.20 & -0.43 & 0.27 & 0.29 & 0.65 & -0.23 & 0.34\\ 0.15 & 0.20 & 0.43 & 0.65 & 0.29 & -0.27 & -0.23 & 0.34\\ 0.15 & 0.20 & 0.43 & -0.65 & 0.29 & 0.27 & -0.23 & 0.34\\ 0.20 & 0.58 & -0.35 & 0 & 0 & 0 & 0.59 & 0.39\\ -0.20 & -0.58 & 0.35 & 0 & 0 & 0 & 0.59 & 0.39\\ -0.64 & 0.28 & 0.09 & 0 & -0.58 & 0 & -0.23 & 0.34\\ 0.64 & -0.28 & -0.09 & 0 & -0.58 & 0 & -0.23 & 0.34 \end{array}\right],$$

(43a)

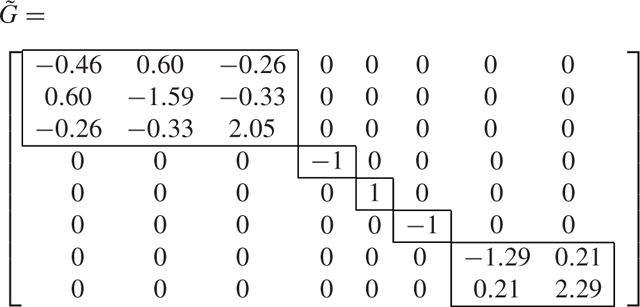




(43b)

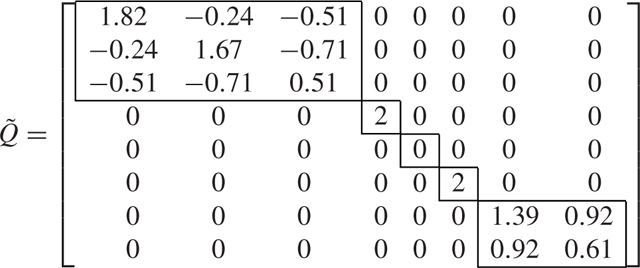



We see that the transformation derived from SBD decomposes the *N* = 8-dimensional problem into one 3-dimensional, one 2-dimensional, and three 1-dimensional problems. Therefore, this example shows that the decomposition using the SBD transformation is finer (the blocks generated are smaller) than the symmetry-based transformation.

### B. EXAMPLE 2: POWERGRID SYSTEM

The purpose of this example is to show that the SBD decomposition can be computed in less time than the symmetry-based decomposition. Here, we use the Irreducible Representation (IRR) code from Ref. [27] and the SBD code from Ref. [47]. Our comparison is applied to real power grid networks of increasing dimension, i.e., with an increasing number of busses. For all the networks that we consider, the equitable and orbital partitions are the same [28], therefore, the inputs provided to both codes are exactly the same, i.e., one adjacency matrix that describes the topology of the network, and indicator matrices that provide information on the clusters to which each node belongs. Table 1 contains information about the selected power grids.

**FIGURE 2.**
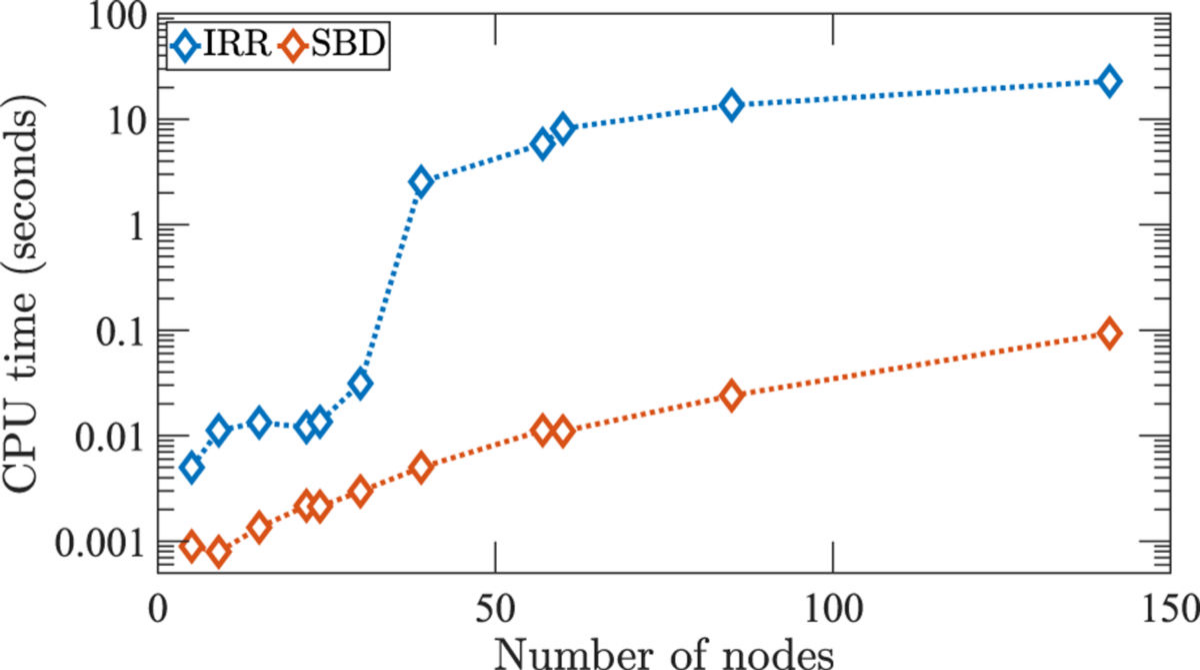
We plot the CPU time to run the SBD code and the IRR code for 11 selected power grids with different numbers of nodes. Additional information on the topology of these networks is provided in Table 1. We see that the SBD algorithm is faster in all cases. As the number of nodes increases, the CPU time for both codes increases, but the CPU time for the IRR algorithm grows more rapidly.

Fig. 2 shows the CPU-time required for running both algorithms (SBD and IRR). We use an Intel Core i7–10700 CPU to run the algorithms. Power grids are chosen such that at least one non-trivial orbital partition (a partition with more than one node in it) is present. The computation complexities associated with the SBD code (Algorithm 1) and the IRR code are $𝓞\left(N^{4}\right)$ and 𝓞(N!), respectively, where *N* is the number of nodes in the network. Fig. 2 shows that the CPU time grows faster as *N* increases for the IRR code compared to the SBD code.

### C. EXAMPLE 3: HVAC SYSTEM

Here we show how the run-time required by the solution of a large-dimensional HVAC optimal control problem is reduced by using the SBD decomposition method. We consider a graph-based HVAC model that was originally introduced in [66]. This model is linear time-invariant and continuous time. It takes the temperature of the walls and zones as states, and the control strategy is to regulate the zones’ temperatures while using the minimum amount of energy. The time evolution of the temperatures is modeled with an RC circuit consisting of lumped thermal resistors and capacitors, whose resistances and capacitances are calculated based on [18]. The procedure to find the linear model is described in Algorithm 1 of [66]. First, an undirected weighted graph is created, for which nodes represent either the walls, the zones, or the ambient. Nodes that are thermally connected share an edge. Connections are between walls and zones, and between the walls and the ambient. Internal walls connect zones and external floor/ceiling walls connect the zones and the ambient. The weight of a connection is the inverse of the thermal resistance between the two connected nodes. We assumed that the resistance of the floor/ceiling walls is greater than that of the internal walls by a factor of 4, and the resistance of the external walls is greater than that of the internal walls by a factor of 1.1. The Laplacian matrix corresponding to the graph of the network formed of walls, zones, and ambient is equal to *L* = *D* − *A* where *A* is the adjacency matrix that describes the topology of the HVAC system, and *D* is a diagonal matrix with entries on the main diagonal equal to the row sums of the matrix *A*.

Next, the continuous time model for the HVAC problem is generated based on the remaining steps of Algorithm 1 from [66]. Discretization of the model is done using a 10 min sampling time. For simplicity, since our focus here is on comparing run-times between the original and the transformed OCP, we assumed that the ambient temperature is 0°*C* and no disturbance is present. Hence, the dynamical equation of the system is,

(44a) $$x_{k+1}=Ax_{k}+Bu_{k},$$

(44b) $$x_{k}=\left[\begin{array}{l} T_{k}^{\text{wall}}\\ T_{k}^{\text{zone}} \end{array}\right]$$

where $T_{k}^{\text{wall}}$ is the vector of the temperatures of all the walls and $T_{k}^{\text{zone}}$ is the vector of the temperatures of all the zones at time *k*. Note that we have added an appropriate number of zero columns to the matrix *B* in order to make it square. The cost function is quadratic in the form,

(45) $$J=\sum_{k=0}^{\infty }x_{k}^{⊤}Qx_{k}+u_{k}^{⊤}Ru_{k}$$

where *Q* is a positive semi-definite diagonal matrix, with entries on the main diagonal that are either one for the zone states, or zero for all the other states. The matrix *R* is a positive definite diagonal matrix, such that $R=\bar{R}⊕I$, where *I* is an identity matrix with appropriate dimension; and for simplicity, we set $\bar{R}$ to be a diagonal matrix with all entries on the main diagonal equal to 0.1. As a result, the original OCP can be written,

(46a) $$\underset{u}{\min}\;J=\sum_{k=0}^{\infty }x_{k}^{⊤}Qx_{k}+u_{k}^{⊤}Ru_{k}$$

(46b) $$\text{s.t.}\;x_{k+1}=Ax_{k}+Bu_{k},\;x(0)=x_{0}.$$

The transformation matrix *T* is equal to,

(47) T=SBD(A,B,Q,R).

and is numerically computed using the SBD algorithm from [46]. This matrix has been applied to decompose the OCP in a similar fashion to Section IV. The complete derivation is provided in Section II of the Supplementary Information. The OCP (46a) is then decomposed in a form similar to (10) with the difference that *l* is used as the superscript here,

(48) $$\underset{v^{l}}{\min}\;J^{l}=\sum_{k=0}^{\infty }z_{k}^{l^{⊤}}{\tilde{Q}}^{l}z_{k}^{l}+v_{k}^{l^{⊤}}{\tilde{R}}^{l}v_{k}^{l}\;\text{s.t.}\;z_{k+1}^{l}={\tilde{A}}^{l}z_{k}^{l}+{\tilde{B}}^{l}v_{k}^{l},\;z^{l}(0)=z_{0}^{l},$$

l=1,…,L.

**FIGURE 3.**
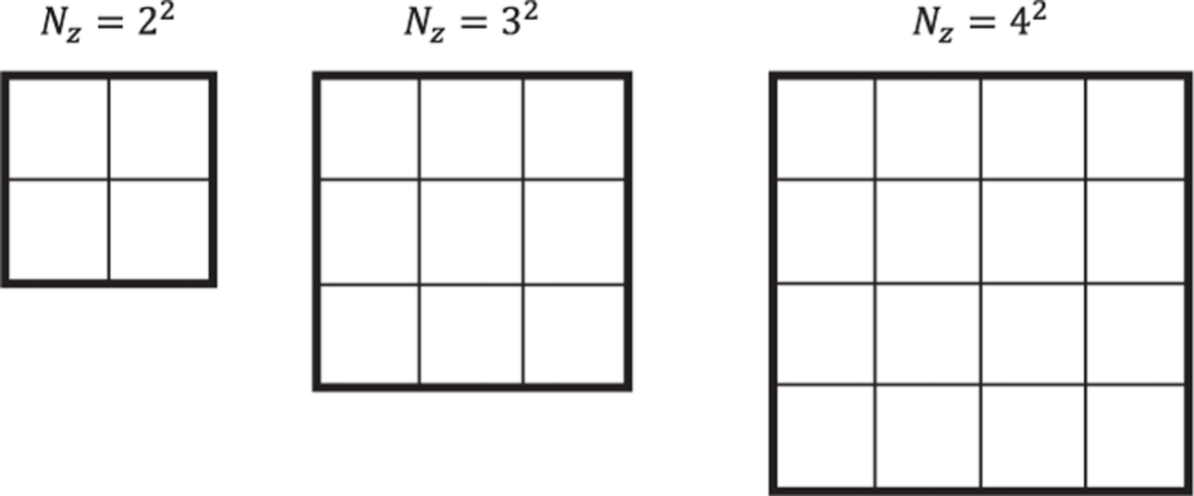
Base structure of the zones and walls for three different cases corresponding to different number of zones: $N_{z}=2^{2}$, $N_{z}=3^{2}$, and $N_{z}=4^{2}$. Narrow black lines indicate the internal walls, and thick black lines are the external walls. Floor/ceiling walls are not shown here.

To investigate the effect of the decompositions on the runtime, we use the base structure for the zones, walls, and ambient, which is shown in Fig. 3, and vary the number of zones, *N*_*z*_. In the analysis that follows, we expand this structure up to *N*_*z*_ = 6^2^.

**FIGURE 4.**
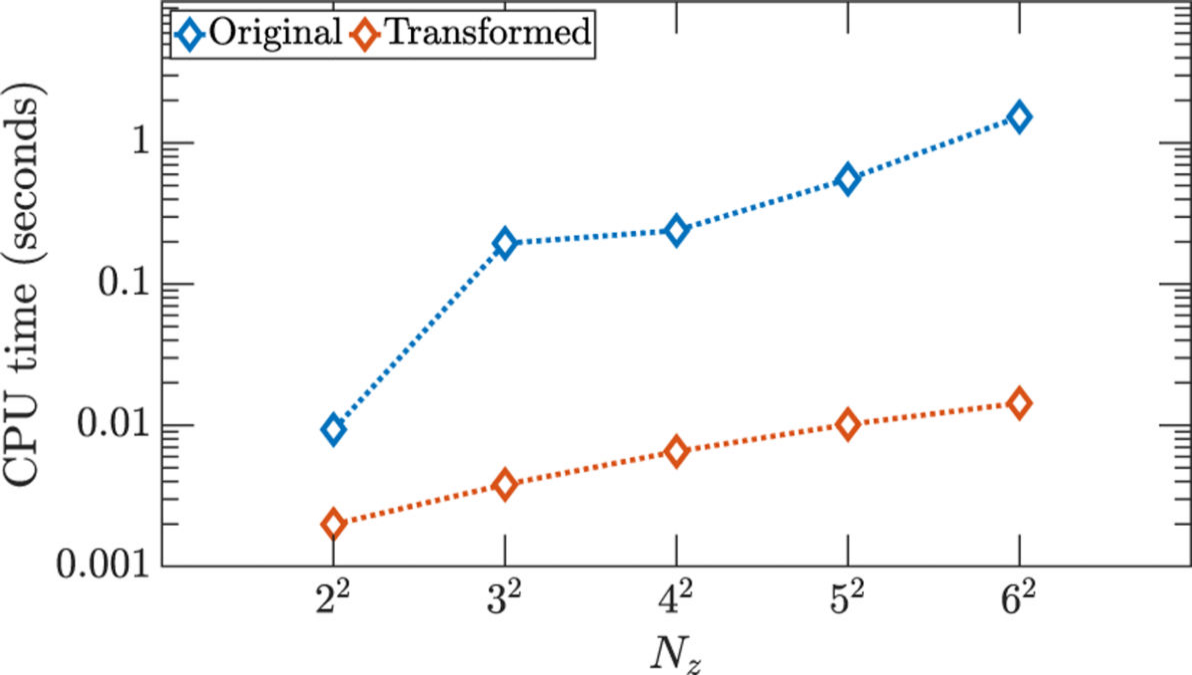
The CPU time for running dlqr Matlab command for the original and transformed OCPs for the HVAC example. Here we plot the CPU time corresponding to the transformed OCP, which is obtained by running the decomposed OCPs in parallel on 16 workers.

**FIGURE 5.**
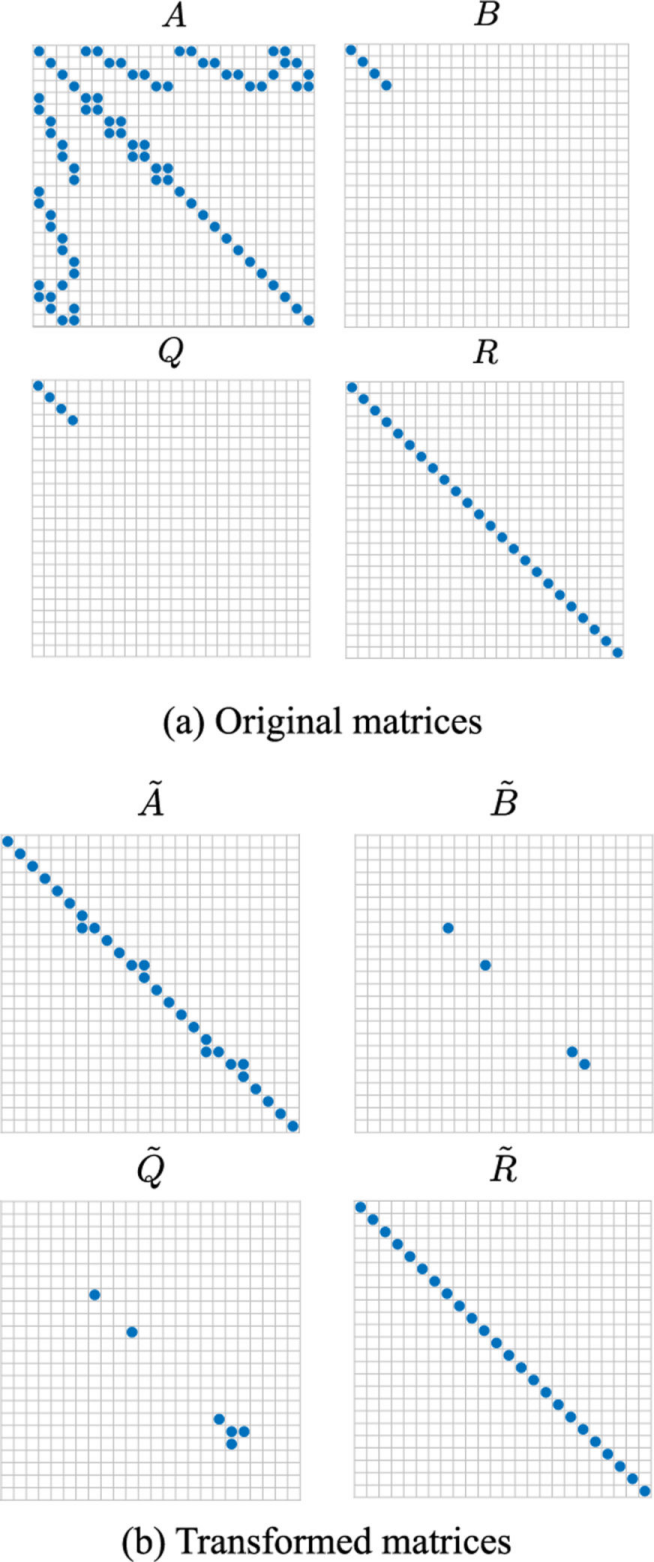
HVAC problem with *N*_*z*_ = 2^2^: comparison between the matrices *A*, *B*, *Q*, and *R* from (46) before and after application of the transformation matrix *T*. The nonzero entries of the original matrices and of the transformed matrices are shown as blue squares. A very good reduction is achieved in this case as the original 24-dimension system is decoupled into 20 subsystems of which 16 have dimension 1 and 4 have dimension 2.

The total number of states is equal to the total number of elements coupled together, i.e., the number of ceiling/floor walls, the number of ambient walls, the number of internal walls, and the number of zones. For the examples in Fig. 3, the number of ceiling/floor walls is equal to $2N_{z}$; the number of ambient walls is equal to $4\sqrt{N_{z}}$; the number of internal walls is equal to $2\left(N_{z}-\sqrt{N_{z}}\right)$; and the number of zones is equal to $N_{z}$. Then the total number of states of the linear discrete-time system is equal to $N_{\text{sys}}=5N_{z}+2\sqrt{N_{z}}$. As a benchmark, we timed the Matlab command dlqr using the Matlab command timeit. The command dlqr finds the optimal feedback gain matrix for the linear quadratic regulator problem in discrete time. This command first solves the algebraic discrete-time Riccati equation and then computes the optimal gain matrix. The average CPU times out of 20 simulations for the original discrete-time OCP and the transformed OCP are shown in Fig. 4. We see from Fig. 4 that the CPU times for the transformed problems are significantly lower than those of the original problem since the decoupled OCPs are lower in dimension and they can be solved using parallel computing. For this particular problem, we were able to break the original large OCP into several smaller OCPs with dimensions equal to or smaller than 2. As an example, in Fig. 5 the matrices *A*, *B*, *Q*, and *R* from (46) for the case with $N_{z}=2^{2}$ are visualized before and after application of the transformation *T*.

We remark that the dimensionality reduction achieved in Fig. 5 is very good as it enables the solution of several OCPs with much reduced computational effort. Table 2 provides detailed information about the dimension of the linear systems, and the number of subsystems of dimension 1 and 2 obtained after the application of the transformation. Overall the table demonstrates the usefulness of the SBD approach in decomposing these OCPs.

We note that in the process of generating the dynamical matrices *A* and *B*, the Laplacian matrix that describes the connectivity of the zones, walls, and the ambient is broken into pieces; see Step 2 of Algorithm 1 in [66]. Since the IRR code [27] requires either the adjacency matrix or the Laplacian matrix as an input, we were unable to use the IRR code to decompose this HVAC problem.

**FIGURE 6.**
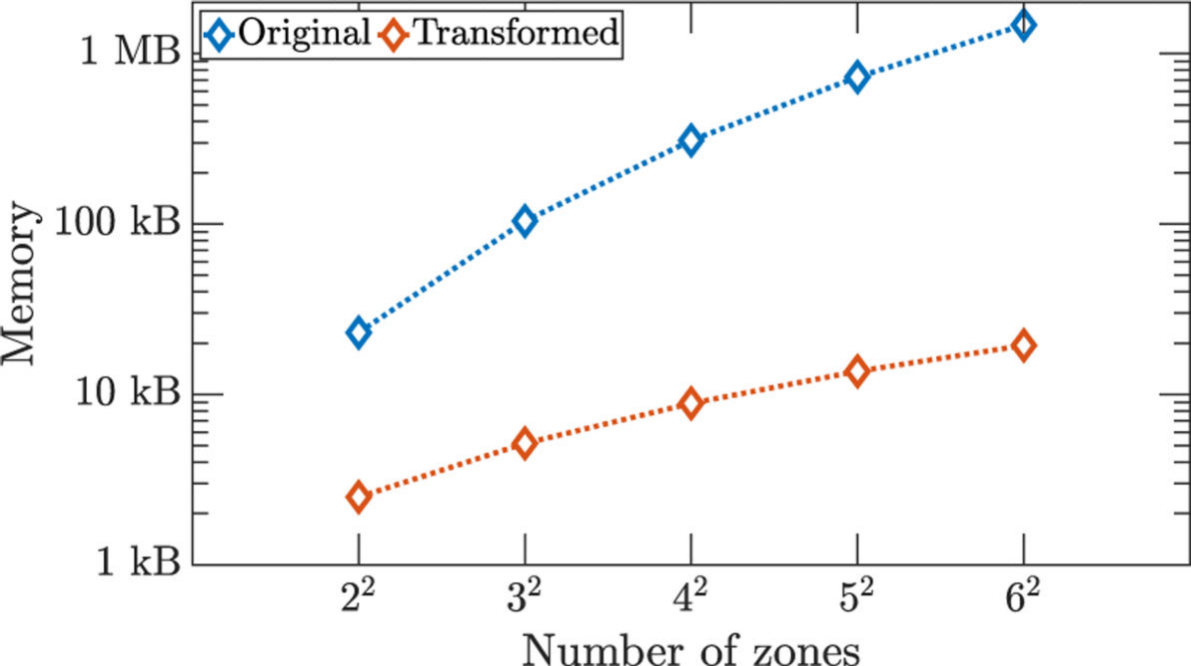
HVAC example. Memory used for storing data when running the dlqr Matlab command for the original and the transformed OCPs. Here we plot the memory in kilobytes corresponding to the transformed OCP, obtained by running the decomposed OCPs in parallel on 16 workers.

In Fig. 6, the memory, in kilobytes (kB), measured by the MATLAB command whos to store data for running dlqr in MATLAB is plotted as the number of zones increases. Since the application of the SBD transformation sets the transformed matrices into a block diagonal form, this results in substantial memory saving, as the dimension of the matrices increases.

## VII. CONCLUSION

In this paper, we have shown that an SBD-based strategy can be conveniently adopted to decouple optimal control problems into lower dimensional problems. These lower dimensional OCPs can then be solved in parallel to achieve faster computation runtimes. In particular, we have shown that the SBD transformation can be used to break the original Riccati equation and the original controllability Gramian into a set of lower-dimensional (and easier to solve) Riccati equations and controllability Gramians, respectively. We have called these reductions ‘exact’ because no information about the original system and the objective function is lost with the decomposition. Therefore, important properties such as stability and the dimension of the controllable and observable subspaces are preserved while the computation time is reduced.

We have used three examples with application to networked systems to demonstrate the advantages of our proposed method when compared to the IRR reduction method. The first example of a simple spring-damper mechanical system demonstrates that the SBD approach decouples the system into lower-dimensional subproblems than the IRR decomposition. In the second example, we have used real data about the topology of several power grid networks of various sizes, and shown that the computation time to compute the SBD transformation is always lower than the IRR method. Finally, our third example combines both these aspects and demonstrates the application of the method to a real-life example; an HVAC control problem.

## Supplementary Material

Supplementary Material

## Figures and Tables

**TABLE 1.** Real power grids. For each power grid network, we report the number of nodes *N*, the number of edges *E*, and the number of non-trivial clusters *C*. All networks are undirected and unweighted.

Name	*N*	*E*	*C*
case5	5	6	2
case9target	9	9	3
casel5nbr	15	14	2
case22	22	21	3
case24_ieee_rts	24	34	1
case30	30	41	1
case39	39	46	3
case57	57	78	1
case60nordic	60	72	4
case85	85	84	10
casel41	141	140	7

**TABLE 2.** Dimensionality reduction for the HVAC systems. The table shows the number of zones *N*_*z*_, the overall dimension of the linear system *N*_sys_, the total number of subsystems obtained after the decomposition $\bar{N}$, the number of subsystems of dimension 1 after the decomposition ${\bar{N}}_{1}$, and the number of subsystems of dimension 2 after the decomposition ${\bar{N}}_{2}$.

N_z_	N_sys_	$\tilde{N}$	${\tilde{N}}_{1}$	${\tilde{N}}_{2}$
2^2^	24	20	16	4
3^2^	51	42	33	9
4^2^	88	72	56	16
5^2^	135	110	85	25
6^2^	192	156	120	36
