# Supplementary material for: Exact Decomposition of Optimal Control Problems via Simultaneous Block Diagonalization of Matrices
Source: IEEE Open J Control Syst. Author manuscript; Available in PMC 2023 Feb 24. (PMC9956949; doi:10.1109/ojcsys.2022.3231553)
Supplement: Supplementary Material [file NIHMS1864919-supplement-Supplementary_Material.pdf]

Received 23 September 2022; revised 18 November 2022; accepted 8 December 2022; date of current version 4 January 2023.
Digital Object Identifier 10.1109/OJCSYS.2022.3231553

Supplementary Information for Exact Decomposition of Optimal Control Problems via Simultaneous Block Diagonalization of Matrices

Amirhossein Nazerian[1] (Graduate Student Member, IEEE), Kshitij Bhatta[2] (Graduate Student Member, IEEE), Francesco Sorrentino[1] (Senior Member, IEEE)

[1] Mechanical Engineering Department, University of New Mexico, Albuquerque, NM 87131 USA
[2] Mechanical and Aerospace Engineering, University of Virginia, Charlottesville, VA 22903 USA

CORRESPONDING AUTHOR: F. Sorrentino (e-mail: fsorrent@unm.edu)

This work is supported by NIH grant 1R21EB028489-01A1.

ABSTRACT We provide further discussion over the sparsity level of the matrices and the extension of the proposed method in the main manuscript to the infinite horizon discrete time optimal control problems.

INDEX TERMS Decoupling, Optimal control, Simultaneous Block Diagonalization.

I. Effects of the sparsity of the input matrices on the performance of Algorithm 1

The most computationally expensive step of Algorithm 1 is step 3 where $\mathrm{vec}(U)$ is found as a vector in the null space of S. If the input matrices $A_1, A_2, \ldots, A_M$ are sparse, the matrix S will also be sparse, which follows from steps 1 and 2. To calculate $\mathrm{vec}(U)$, one can simply use the sparsity routines available in many programming languages; thus, we expect to see a smaller run-time of the code as the level of sparsity is increased for a fixed dimension of the matrices.

As an example, we have generated $n = 70$ node undirected and unweighted Erdös-Rényi graphs (random graphs) by varying the connection probability p, i.e., the probability with which any two nodes i and $j \neq i$ are connected. For each value of $p \in [0.1, 0.8]$, we have generated 20 graphs and provided the adjacency matrix A corresponding to the generated graph as the input to the SBD code generated from Algorithm 1 in the main manuscript. We have plotted the average run-time of the SBD cod as p is varied. The standard deviations are shown as colored backgrounds. Simulations were performed on the same PC as in Sec, VI of the main manuscript. We see that as the adjacency matrix becomes more sparse (as p decreases), the run-time decreases too. We conclude that the SBD code benefits from the sparsity structure of the input matrices.

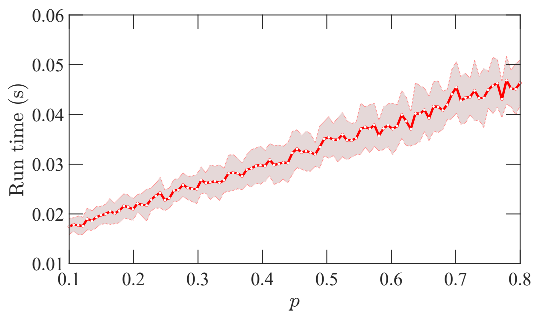

FIGURE 1: The average run-time of the SBD code based on Algorithm 1 of the main manuscript as the connection probability p is varied. The standard deviation of the data plotted is shown as a colored background. We see from the plot that as the matrices become more sparse, the run-time decreases.

II. Infinite Horizon Discrete-time OCP

Consider the infinite horizon discrete-time OCP,

$$\min_{\boldsymbol{u}_k} \; J = \frac{1}{2} \sum_{k=0}^{\infty} \left(\boldsymbol{x}_k{}^\top Q \boldsymbol{x}_k + \boldsymbol{u}_k{}^\top R \boldsymbol{u}_k + 2 \boldsymbol{x}_k{}^\top F \boldsymbol{u}_k \right), \quad \text{(1a)}$$

$$\text{s.t.} \quad \boldsymbol{x}_{k+1} = A \boldsymbol{x}_k + B \boldsymbol{u}_k, \; \boldsymbol{x}_k = \boldsymbol{x}_0, \quad \text{(1b)}$$

where the matrices $A, B, Q, R, F \in \mathbb{R}^{n \times n}$, $Q \succeq 0$, and $R \succ 0$, $Q - FR^{-1}F^\top \succeq 0$, and the vectors $\boldsymbol{x}_k, \boldsymbol{u}_k \in \mathbb{R}^n$. Under

This work is licensed under a Creative Commons Attribution 4.0 License. For more information, see https://creativecommons.org/licenses/by/4.0/

the assumption that the pair (A, B) is controllable and that the pair $(A - BR^{-1}F^\top, Q - FR^{-1}F^\top)$ is observable, a unique solution to (1) exists. We note that if the length of $\boldsymbol{u}$ is not equal to n, the dimension of the matrices can be adjusted similarly to (4) from the main manuscript.

The original large OCP is decoupled into a set of L lower dimensional problems by simultaneously block diagonalizing A,B,Q,R, and F, using the orthogonal similarity transformation matrix T. The transformed matrices $\tilde{A}, \tilde{B}, \tilde{Q}, \tilde{R}, \tilde{F}$ are decomposed as in (6) of the main manuscript and every block l of each matrix has the same size n_l, $\sum_{l=1}^{L} n_l = n$.

By pre-multiplying (1b) by $T^\top$, defining $\boldsymbol{z}_k := T^\top \boldsymbol{x}_k$ and $\boldsymbol{v}_k := T^\top \boldsymbol{u}_k$, the dynamics and cost can be written in terms of the new variable $\boldsymbol{z}_k$. So, the transformed OCP is

$$\min_{\boldsymbol{z}_k} \ J = \frac{1}{2} \sum_{k=0}^{\infty} \left(\boldsymbol{z}_k{}^\top \tilde{Q} \boldsymbol{z}_k + \boldsymbol{v}_k{}^\top \tilde{R} \boldsymbol{v}_k + 2 \boldsymbol{z}_k{}^\top \tilde{F} \boldsymbol{v}_k \right), \tag{2a}$$

$$\text{s.t.} \quad \boldsymbol{z}_{k+1} = \tilde{A} \boldsymbol{z}_k + \tilde{B} \boldsymbol{v}_k, \ \boldsymbol{z}_0 = \boldsymbol{z}^0. \tag{2b}$$

The optimal solution is,

$$\boldsymbol{u}_k^* = -R^{-1}(B^\top \boldsymbol{\lambda}_{k+1}^* + F^\top \boldsymbol{x}_k^*), \tag{3}$$

where $\boldsymbol{\lambda}_{k+1} = P\boldsymbol{x}_{k+1}$ and the matrix P can be found by solving the algebraic discrete-time Riccati equation,

$$\begin{aligned} -(A^\top PB + F)(B^\top PB + R)^{-1}(B^\top PA + F^\top) \\ + A^\top PA - P + Q = 0 \end{aligned} \tag{4}$$

The rest of the analysis for the case of the infinite time discrete-time OCPs is similar to what is described in Sec. IV of the main manuscript.
